# Maternal high-fat diet exposure is associated with altered hypothalamic microglial development and reduced early postnatal TGFβ1 signaling in male offspring

**DOI:** 10.3389/fendo.2026.1862234

**Published:** 2026-06-22

**Authors:** Nan Chen, Huabin Ye, Yi Ren

**Affiliations:** Department of Geriatrics, The First Affiliated Hospital of Shenzhen University, Shenzhen Second People’s Hospital, Shenzhen, China

**Keywords:** homeostatic-like microglial features, hypothalamic microglia development, maternal obesity, Parkin, TGFβ1/SMAD signaling

## Abstract

**Background:**

The hypothalamus plays a central role in maintaining energy homeostasis by integrating peripheral metabolic signals. Maternal high-fat diet (HFD) exposure has been shown to disrupt hypothalamic development and increase offspring susceptibility to metabolic disorders. Microglia, as key regulators of neuroimmune interactions and synaptic remodeling, are increasingly recognized as critical mediators of metabolic programming. During early postnatal development, microglia undergo a transition toward a homeostatic phenotype characterized by markers such as TMEM119, a process that is critically dependent on canonical TGFβ1/SMAD3 signaling. However, whether maternal HFD exposure interferes with the acquisition of homeostatic-like microglial features during this sensitive developmental window remains unclear.

**Methods:**

We characterized the postnatal trajectory of hypothalamic microglia-associated developmental features using single-cell RNA sequencing and temporally resolved immunohistochemical analyses in male offspring of HFD–fed dams. Microglial abundance, morphology, and expression of homeostatic markers were assessed across developmental stages in the mediobasal hypothalamus (MBH). Canonical TGFβ1 signaling activity was evaluated by pSMAD3 immunoreactivity, and Parkin-associated signals were quantified within hypothalamic tissue and microglia-defined regions of interest. To assess whether restoration of local TGFβ1 signaling influences microglia-associated developmental features, neonatal MBH supplementation with recombinant TGFβ1 was performed.

**Results:**

Single-cell transcriptomic analysis revealed that hypothalamic microglia progressively acquired homeostatic-associated transcriptional features during early postnatal development, with increasing enrichment of homeostatic marker-associated signatures by the juvenile stage. Maternal HFD exposure attenuated the developmental increase of TMEM119^+^ cells in the MBH and was associated with altered microglial abundance and morphology across postnatal stages. During a critical early postnatal window, male offspring born to HFD-fed dams exhibited reduced pSMAD3 immunoreactivity in IBA1^+^ microglia and decreased Parkin-associated signal in both hypothalamic tissue and microglial regions. Neonatal TGFβ1 supplementation was associated with increased pSMAD3 immunoreactivity in IBA1-positive cells, increased Parkin-associated signal within standardized IBA1-centered regions of interest, and increased TMEM119-positive cell abundance at later juvenile stages.

**Conclusion:**

These findings support a developmental association between maternal HFD exposure, reduced early postnatal TGFβ1/SMAD3 signaling within the MBH, and altered acquisition of homeostatic-like microglial features in the hypothalamus. These developmental neuroimmune alterations may contribute to increased susceptibility to later metabolic dysfunction in male offspring.

## 1 Introduction

The hypothalamus is a central integrator of energy homeostasis, orchestrating whole-body energy balance by processing peripheral metabolic cues (1, 2). Accumulating evidence suggests that early-life exposure to maternal obesity disrupts the structural and functional development of the hypothalamus (3). Underlying mechanisms include increased susceptibility to endoplasmic reticulum stress and proteostasis disturbances (4, 5), altered neuronal subtype specification during neurogenesis (6) and disrupted synaptic circuit formation. These neurodevelopmental disturbances are thought to predispose offspring to obesity and other metabolic disorders, particularly in calorie-rich environments.

Microglia have emerged as critical regulators in the developmental programming of metabolic homeostasis. As the brain’s resident immune cells, they modulate hypothalamic neuroinflammation, synaptic plasticity, and neuronal activity that underlie energy regulation (7). In rodent models, microglia in the arcuate nucleus actively mediate synaptic pruning, shaping POMC/AgRP circuits that govern feeding behavior and energy expenditure (8). Exposure to maternal HFD or gestational inflammation induces epigenetic and developmental reprogramming of microglia (9, 10), resulting in impaired leptin signaling, aberrant synaptic development, and altered behavioral outcomes (11–13).

Postnatally, microglia undergo a transition from a highly reactive and phagocytic state to a ramified phenotype that underlies their homeostatic functions (14). One characteristic feature accompanying this developmental transition is the progressive upregulation of TMEM119, a microglia-specific marker that is absent in peripheral immune cells and frequently co-expressed with P2RY12. TMEM119^+^/P2RY12^+^ microglia are widely considered characteristic markers of homeostatic microglial identity and are associated with maintenance of CNS immune homeostasis and restraint of excessive inflammatory activation (15). Importantly, Tmem119^-^/^-^ mice exhibit exaggerated inflammatory responses and pronounced neuronal injury upon lipopolysaccharide (LPS) exposure, highlighting the neuroprotective role of this homeostatic microglial subpopulation (16). The acquisition of homeostatic-like microglial features is critically regulated by transforming growth factor-β1 (TGF-β1) signaling, particularly during the first two postnatal weeks, a sensitive developmental window for the CNS (17). Within microglia, TGF-β1 acts in a cell-autonomous manner to activate TGF-β receptors and trigger canonical SMAD-dependent signaling cascades, which in turn initiate transcriptional programs essential for microglial maturation (18, 19). Disruption of this pathway hampers the establishment of mature microglial morphology and homeostatic gene expression, resulting in persistently activated microglia that compromise normal brain development and may perturb hypothalamic circuits involved in energy homeostasis (12, 20).

While previous studies have established a link between maternal obesity and widespread microglial activation (21), direct evidence addressing its impact on the early postnatal acquisition of homeostatic-like features in hypothalamic microglia remains scarce. Notably, it is still unclear whether adverse maternal environments perturb the developmental trajectory of this regulatory microglial subpopulation before the onset of metabolic dysfunction. To address this gap, the present study systematically characterizes the temporal dynamics of homeostatic-like microglial features and associated signaling pathways in male offspring born to dams fed a high-fat diet, and further explores early postnatal interventions aimed at supporting microglial homeostatic features and preserving hypothalamic energy-regulatory integrity.

## 2 Materials and methods

### 2.1 Animals and treatment

The animal research was performed in compliance with the National Institutes of Health guide for the care and use of laboratory animals, and was approved by the Animal Care and Management Committee of the Shenzhen Second People’s Hospital (Approval No- 202300107 and 202400151).

#### 2.1.1 Dams

Female C57BL/6J wild-type mice were maintained under specific pathogen–free (SPF) conditions (five per cage) in a temperature-controlled room with a 12-h light/12-h dark cycle. At five weeks of age, females were randomly assigned to receive either a low-fat diet (LFD; 10% kcal from fat; Research Diets, D12450B) or a high-fat diet (HFD; 45% kcal from fat; Research Diets, D12451) and were maintained on the assigned diet for five weeks prior to breeding. For maternal metabolic characterization, 30 dams per dietary group were initially included for HFD or LFD exposure. Subsequently, females were paired with age-matched C57BL/6J males maintained on LFD, and successful mating was confirmed by the presence of a vaginal plug. Dams remained on their respective diets throughout gestation and lactation. The day of delivery was designated as postnatal day 0 (P0). Within 48 h after birth, liters were standardized to six pups per dam, ensuring an approximately balanced sex ratio. This maternal HFD exposure model generated male offspring cohorts for downstream analyses of hypothalamic microglial development across early postnatal stages.

#### 2.1.2 Offspring

Male offspring born to dams maintained on LFD or HFD exposure throughout preconception, gestation, and lactation periods were hereafter referred to as L-o and H-o, respectively. To minimize sex-related variability—and based on previous reports indicating that maternal obesity induces earlier and more pronounced metabolic abnormalities in male offspring (22), only males were included in this study. After weaning (postnatal day 21, P21), male offspring were group-housed (five per cage) under SPF conditions and maintained on an LFD until six weeks of age. Brains were collected following transcardial perfusion at P8, P10, P14, P28, P35, and P42 from both groups. Body weight was measured daily before weaning and weekly thereafter.

#### 2.1.3 Neonatal MBH microinjection

At P0, male H-o pups were identified based on anogenital distance and briefly anesthetized by hypothermia prior to stereotaxic procedures. Pups from independent liters were randomly assigned to injection conditions while preserving litter-level biological independence. Neonatal stereotaxic procedures were adapted from previously described protocols for neonatal mouse brain microinjection. Because the mediobasal hypothalamus (MBH) is anatomically deeper than the hippocampal or striatal regions targeted in previous neonatal study (23), preliminary pilot experiments were performed using trypan blue injections at multiple stereotaxic coordinates to empirically optimize MBH targeting. Based on these validation experiments, the following coordinates were selected as providing the most consistent localization within the neonatal MBH: AP + 0.65 mm from lambda, ML ±0.50 mm, DV −4.00 mm. Recombinant human TGFβ1 (Sino Biological, 10804-HNAC) was diluted to 0.1 μg/mL in artificial cerebrospinal fluid (aCSF) and bilaterally infused into the MBH (1 μL per side), resulting in a localized delivery dose of 0.1 ng per side. Control L-o and H-o mice received equivalent bilateral aCSF injections. The selected TGFβ1 concentration was guided by previously reported cerebrospinal fluid and hypothalamic TGFβ1 levels in chow-fed adult mice (24) and neonatal rats (25), together with preliminary optimization experiments performed during development of the neonatal MBH injection protocol. Based on these considerations, 0.1 μg/mL was selected for subsequent experiments. All pups were returned to their dams immediately after injection and maintained with their respective dams until weaning. After weaning, male offspring were transitioned to a LFD and maintained until six weeks of age for downstream analyses. Because treatment allocation was determined at the time of neonatal intracerebral injection, blinding was not feasible during the injection procedure itself. However, image acquisition, quantitative image analysis, and statistical analyses were performed with investigators blinded to treatment group allocation. Direct assessment of TGFβ1 diffusion beyond the targeted MBH region was not performed. Regional specificity was inferred from the localized increase in pSMAD3 immunoreactivity observed within the MBH following TGFβ1 administration.

### 2.2 Immunofluorescence staining

Following transcardial perfusion, brains were cryosectioned into serial 20µm coronal sections. Multiplex IF staining was performed using a TSA-based kit (Abclonal Technology, Cat#RK05903) according to the manufacturer’s instructions. After heat-induced antigen retrieval in citrate buffer (pH 9.0), sections were incubated with hydrogen peroxide for 15 min in the dark, rinsed in PBS, and blocked for 1 h at room temperature in PBS containing 0.3% Triton X-100 and 10% goat serum. Primary antibodies were applied overnight at 4 °C, followed by PBS washes and incubation with an HRP-conjugated anti-rabbit secondary antibody for 30 min at room temperature. After additional PBS washes, sections were incubated with fluorophore-conjugated tyramide reagents (TYR-520, TYR-570, or TYR-690) for 15 min in the dark. For multiplex rounds, sections underwent antigen retrieval to enable the subsequent staining cycle. After the final round, sections were coverslipped using an anti-fade mounting medium containing DAPI (Sigma-Aldrich, Cat#F6057). The following primary antibodies were used for multiplex immunofluorescence staining: rabbit anti-IBA1 (1:500, Wako, Cat#019-19741), rabbit anti-Parkin (1:200, ZEN BIO, Cat#R381626), and rabbit anti-phospho-SMAD3 (1:300, Abcam, Cat#ab52903). For single-label TMEM119 staining, sections underwent heat-induced antigen retrieval, were allowed to cool to room temperature, and were blocked using the same blocking solution for 1 h at room temperature. Sections were then incubated overnight at 4 °C with rabbit anti-TMEM119 (1:200, Cell Signaling Technology, Cat# 90840S), followed by incubation with Goat Anti-Rabbit IgG H&L Alexa Fluor^®^ 555 (1:500, Invitrogen, Cat#A-21428) for 30 min at room temperature. Sections were finally coverslipped using an anti-fade mounting medium containing DAPI.

### 2.3 Image analysis

Images of the MBH were acquired using a ZEISS LSM 800 confocal microscope equipped with 10× and 63× objectives. Imaging parameters were selected based on the specific analytical purpose of each experiment. Low-magnification images were used for quantification of region-wide signal distribution within the MBH, including analyses of IBA1^+^, TMEM119^+^, and IBA1^+^/pSMAD3^+^ cell abundance. In contrast, higher-magnification images were acquired specifically for analyses requiring single-cell resolution, including microglial morphology measurements and quantification of Parkin-associated puncta within standardized IBA1-centered regions of interest (ROI). The MBH was delineated based on neuroanatomical landmarks of the anterior tuberal hypothalamus, encompassing the arcuate nucleus (ARC), ventromedial hypothalamus (VMH), and dorsomedial hypothalamus (DMH). For regional quantification, three to five anatomically matched coronal sections were analyzed per animal, and averaged prior to litter-level aggregation. Z-stack images were collected to reconstruct the three-dimensional architecture of the MBH, and maximum intensity projections were generated for quantitative analysis. This approach was chosen to capture overall pathway activation and signal distribution across the tissue rather than subcellular localization patterns. Quantification of IBA1^+^ microglial process length was performed using the Neuroanatomy plugin in ImageJ. For Parkin puncta analysis, a standardized ROI (100 µm²) was defined around each IBA1^+^ cell, and the number of puncta within each ROI was calculated. Notably, this analysis was designed to quantify relative puncta abundance within individual microglia and was not intended as a colocalization assessment between IBA1 and Parkin signals. The relative proportion of specific cell populations was normalized to the total number of DAPI^+^ nuclei within the corresponding tissue section. Image acquisition and downstream quantitative analyses were performed by investigators blinded to experimental group identity and developmental stage wheneverpossible.

### 2.4 Single-cell RNA sequencing data processing

Publicly available single-cell RNA sequencing (scRNA-seq) data were obtained from the GEO database (accession number GSE132355). We utilized the log2-CPM expression matrix provided by the authors (GSE132355_E10-P45_log2cpm.rds) and focused on five postnatal and developmental stages (E18, P4, P8, P14, and P45). A Seurat object was constructed directly from the supplied log2-CPM values. Because the input dataset consisted of preprocessed log2-CPM expression values rather than raw count matrices, read-level quality control and per-cell filtering based on metrics such as nCount_RNA, nFeature_RNA, and the proportions of mitochondrial, ribosomal, or hemoglobin genes were not re-applied. In addition, standard normalization procedures for raw-count single-cell datasets (e.g., NormalizeData or SCTransform) were not performed. All downstream analyses were conducted directly on the stage-filtered log2-CPM expression matrix. Highly variable genes were re-identified within the processed dataset using the variance-stabilizing transformation (VST) method implemented in Seurat, followed by PCA, UMAP visualization, graph-based clustering, and marker enrichment analyses. Accordingly, the single-cell analyses were primarily intended to provide a developmental reference framework and relative marker-enrichment patterns across developmental stages rather than absolute transcript quantification.

### 2.5 Marker identification and cell-type annotation

Highly variable genes were identified using Seurat’s FindVariableFeatures function (method = “vst”, 2,000 genes), followed by data scaling with ScaleData. Principal component analysis (PCA) was then performed, and Uniform Manifold Approximation and Projection (UMAP) embeddings were generated using the first 20 principal components (PCs) (RunUMAP, dims = 1:20). A shared nearest-neighbor (SNN) graph was constructed on the same 20 PCs (FindNeighbors), and unsupervised clustering was performed using the Louvain algorithm across a range of resolution parameters (0.01, 0.05, 0.1, 0.2, 0.3, 0.5, 0.8, and 1.0). Cluster markers were identified with Seurat’s FindAllMarkers function (Wilcoxon rank-sum test; only.pos = TRUE; min.pct = 0.25; logfc.threshold = 0.25), with multiple-testing correction automatically applied by Seurat. At the selected resolution (0.3), clusters were manually annotated based on canonical marker expression and global transcriptional profiles, yielding the following cell-type identities: neural progenitor cells (NPCs), neural stem cells (NSCs), neurons, immature neurons, ependymocytes, oligodendrocyte precursor cells (OPCs), microglia, oligodendrocytes, astrocytes, endothelial cells, fibroblasts, vascular smooth muscle cells (VSMCs).

### 2.6 Microglial subcluster analysis

Microglial cells were extracted from the annotated hypothalamic dataset for dedicated subclustering. Within this subset, highly variable genes were re-identified and used for dimensionality reduction via PCA and UMAP based on the first 20 PCs. Unsupervised clustering was performed using the Louvain algorithm across multiple resolution parameters, with a resolution of 0.05 selected based on cluster stability and marker gene expression profiles. Marker-based analysis delineated five transcriptionally distinct microglial subtypes: progenitor-like, homeostatic marker-enriched, activated-like, anti-inflammatory marker-enriched, and perivascular-like microglia. To examine developmental trajectories, stage-wise proportions of each subtype (E18, P4, P8, P14, and P45) were quantified, revealing dynamic shifts in microglial composition across postnatal development.

### 2.7 Hypothalamus bulk RNA sequencing data processing and gene expression quantification

Gene-level raw count data for the hypothalamus were obtained from the GEO database (accession number GSE162744). Six predefined samples were retained for analysis. Gene IDs served as row identifiers, and gene lengths were aligned across samples; entries with missing or zero length were excluded. Transcripts per million (TPM) values were computed by first deriving reads per kilobase (RPK), followed by rescaling using each sample’s per-million normalization factor. Genes with counts > 1 in at least two samples were retained for subsequent analyses. Quality control was conducted on log2(TPM + 1) data using (i) distributional boxplots, (ii) sample-to-sample distance heatmaps with hierarchical clustering, and (iii) principal component analysis (PCA). ENSEMBL gene identifiers were mapped to gene symbols using the org.Mm.eg.db annotation package, and duplicated mappings were collapsed to unique symbols. Lowly expressed genes were filtered using edgeR’s filterByExpr function based on the experimental design. Differential expression analysis (DEA) was performed with DESeq2 on the raw count matrix, including size-factor normalization, gene-wise dispersion estimation, and Wald tests with Benjamini–Hochberg false discovery rate (FDR) correction. Differentially expressed genes (DEGs) were defined at adjusted P < 0.05. Upregulated and downregulated gene sets were subsequently subjected to Gene Ontology (GO) enrichment analysis. To identify potential regulatory drivers within upstream or downstream pathways, the gene lists associated with each significant GO term were parsed, and the frequency of gene occurrence across enriched terms was quantified separately for up- and downregulated sets.

### 2.8 Statistics

Normality of all datasets was assessed using the Shapiro–Wilk test prior to statistical analyses. Because all datasets passed the normality test (P > 0.05), data are presented as mean ± standard error (SEM) and were analyzed using parametric statistical methods. Statistical analyses were performed using GraphPad Prism (version 8.0). For maternal metabolic phenotypes, including maternal body weight and glucose tolerance test (GTT) analyses, each data point represented an individual dam. For offspring phenotypes, the litter rather than the individual offspring was treated as the biological statistical unit to ensure the independence of biological replicates. When multiple pups from the same litter were analyzed, pup-level measurements were averaged to generate a single litter-level biological value prior to statistical testing. Unless otherwise specified, developmental and histological analyses were performed using six independent liters per experimental group and developmental stage. Comparisons between two independent groups were evaluated using unpaired two-tailed Student’s *t*-tests. For datasets involving more than two groups, one-way analysis of variance (ANOVA) followed by Tukey’s multiple-comparison test was applied. Longitudinal body weight trajectories were analyzed using repeated-measures two-way ANOVA. Developmental histological measurements (TMEM119^+^, IBA1^+^, IBA1^+^/pSMAD3^+^, and related parameters) were analyzed using ordinary two-way ANOVA with maternal diet and postnatal age as independent factors, followed by Sidak’s multiple-comparisons test. A *p*-value < 0.05 was considered statistically significant.

## 3 Results

### 3.1 Postnatal establishment of hypothalamic microglia and acquisition of homeostatic-like features

**Figure 1:**
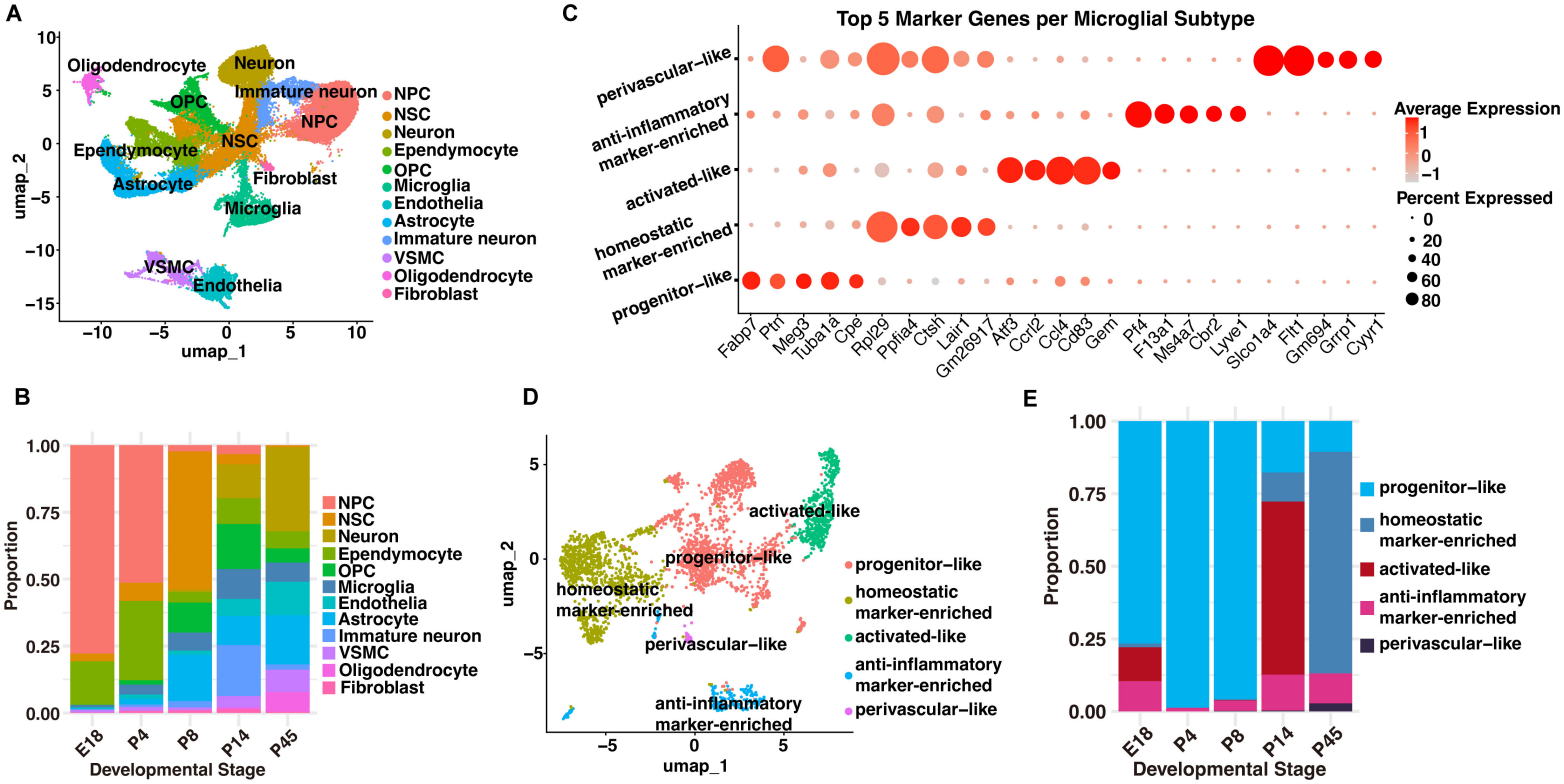
Developmental trajectory of hypothalamic microglia during postnatal development. **(A)** UMAP visualization of single-cell RNA sequencing data from the wild-type mouse hypothalamus across five developmental stages (E18, P4, P8, P14, and P45), identifying 12 distinct cell clusters. **(B)** Temporal changes in the relative proportions of the 12 identified clusters across developmental stages. **(C)** Subclustering of the microglial population into five transcriptionally distinct subtypes based on their top marker genes. **(D)** UMAP plot showing the spatial distribution of the five microglial subpopulations. **(E)** Temporal dynamics of the relative proportions of the five microglial subtypes during postnatal development.

We analyzed single-cell RNA sequencing data from the hypothalamus of wild-type mice across developmental stages. Dimensionality reduction and clustering identified 12 distinct cell clusters based on molecular markers (**Figure 1A**). Microglia were already present at embryonic day 18 (E18) and reached their peak proportion at postnatal day 14 (P14), thereafter maintaining a relatively stable abundance into the juvenile stage (**Figure 1B**). Subclustering of the microglial population revealed five transcriptionally distinct subpopulations, each defined by unique top marker genes (**Figure 1C**). These subclusters were annotated as progenitor-like, homeostatic marker-enriched, activated-like, anti-inflammatory marker-enriched, and perivascular-like microglia, with progenitor-like, homeostatic marker-enriched, and activated-like subtypes being the most prevalent (**Figure 1D**). The top 10 marker genes used for subcluster annotation were provided in **Supplementary Table 1**. Temporal distribution analysis demonstrated that progenitor-like microglia predominated before P8, whereas from P14 onward, activated-like microglia became increasingly prominent, coinciding with the emergence of homeostatic marker-enriched microglia. By the juvenile stage, homeostatic marker-enriched microglial populations became increasingly prominent, accompanied by smaller proportions of anti-inflammatory marker-enriched and perivascular-like subtypes (**Figure 1E**).

### 3.2 Maternal HFD exposure is associated with altered postnatal TMEM119-positive cell abundance within the hypothalamus

**Figure 2:**
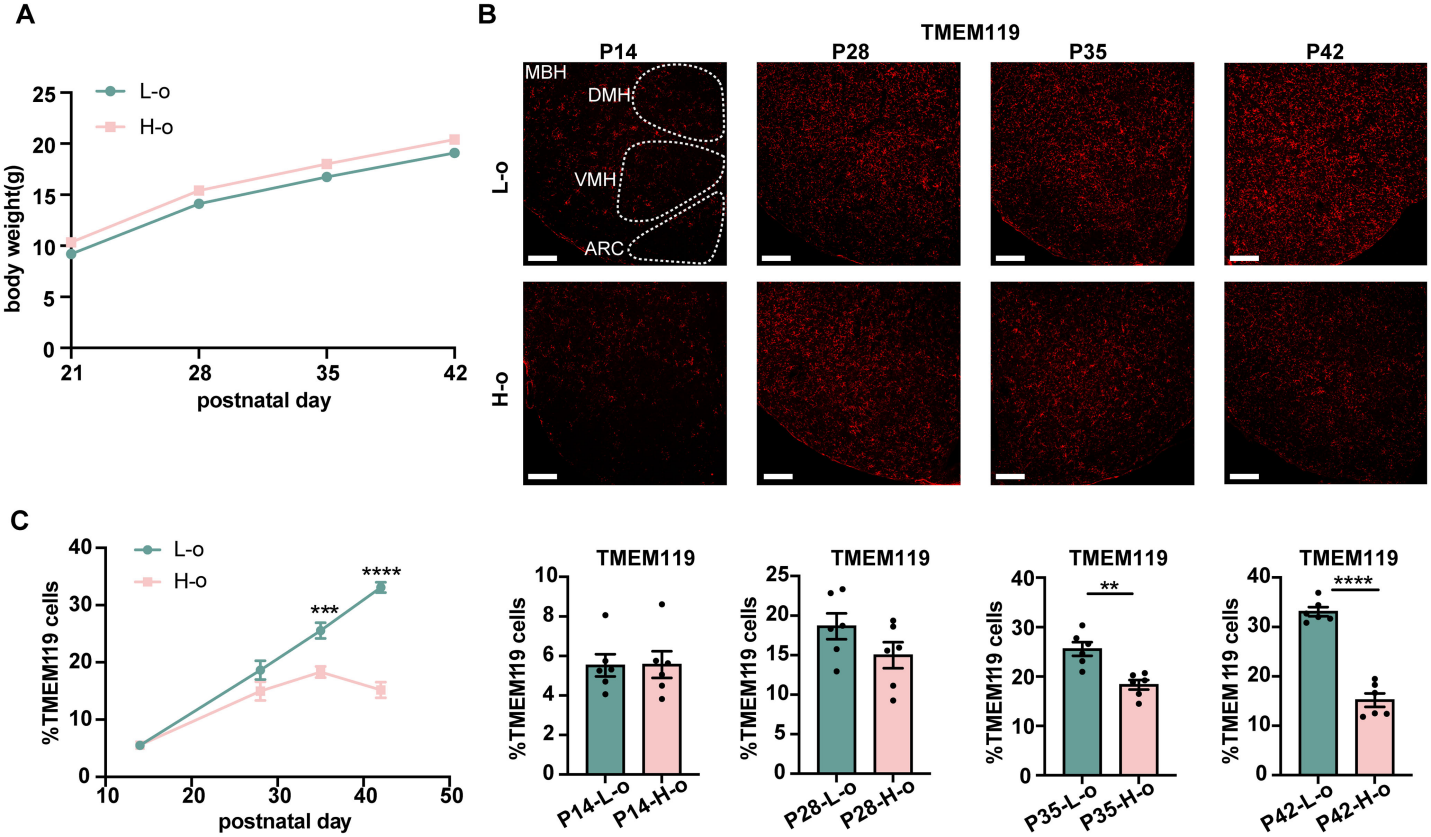
Reduced TMEM119-positive cell abundance within the MBH of male offspring born to HFD-fed dams. **(A)** Body weight (BW) trajectories from weaning to the juvenile stage in male offspring born to dams fed a low-fat diet (L-o) or a high-fat diet (H-o). Each data point represented the mean ± SEM BW per litter (L-o: n = 10 liters; H-o: n = 10 liters). **(B)** Representative low-magnification immunofluorescence images showing the regional distribution of TMEM119^+^ cells across the mediobasal hypothalamus (MBH) at P14, P28, P35, and P42 in L-o and H-o groups. These images were intended to illustrate region-wide and population-level signal patterns rather than single-cell morphology. Scale bar, 150 µm. **(C)** Quantification of the proportion of TMEM119^+^ cells normalized to total DAPI^+^ nuclei within the MBH at P14, P28, P35, and P42. Each data point represented one litter-level biological replicate (n = 6 liters per group). Data were presented as mean ± SEM. Statistical analyses were performed using unpaired two-tailed Student’s t test or two-way ANOVA followed by Sidak’s multiple-comparisons test. **p < 0.01, ***p < 0.001, ****p < 0.0001.

Short-term maternal HFD exposure for 5 weeks was sufficient to induce significant body weight gain in female mice (**Supplementary Figure 1A**) and impaired glucose tolerance (**Supplementary Figure 1B**) prior to mating. Consistent with our previous studies demonstrating pronounced postnatal catch-up growth in male offspring born to HFD-fed dams (5, 26), we similarly observed accelerated body weight gain in H-o offspring from birth to weaning in the current cohort (**Supplementary Figure 1C**). Subsequently, we monitored body weight trajectories of both groups of offspring maintained on a LFD from weaning through the juvenile stage. No significant differences in body weight were observed between the two groups during early juvenile development (**Figure 2A**). To investigate postnatal changes in TMEM119^+^ cells abundance during the same postnatal period, we examined TMEM119^+^ expression in the MBH of offspring at P14, P28, P35, and P42 (**Figure 2B**). In L-o mice, the proportion of TMEM119^+^ cells within the MBH increased progressively from P14 to P42 (**Figure 2C**). In contrast, H-o exhibited a markedly slower increase beginning at P14 (**Figure 2C**), with a significant reduction at P35 (*p* = 0.0016) and a further decrease by P42 (*p* < 0.0001) compared with L-o controls (**Figure 2D**).

### 3.3 Maternal HFD exposure induces morphological alterations of hypothalamic microglia in male offspring

**Figure 3:**
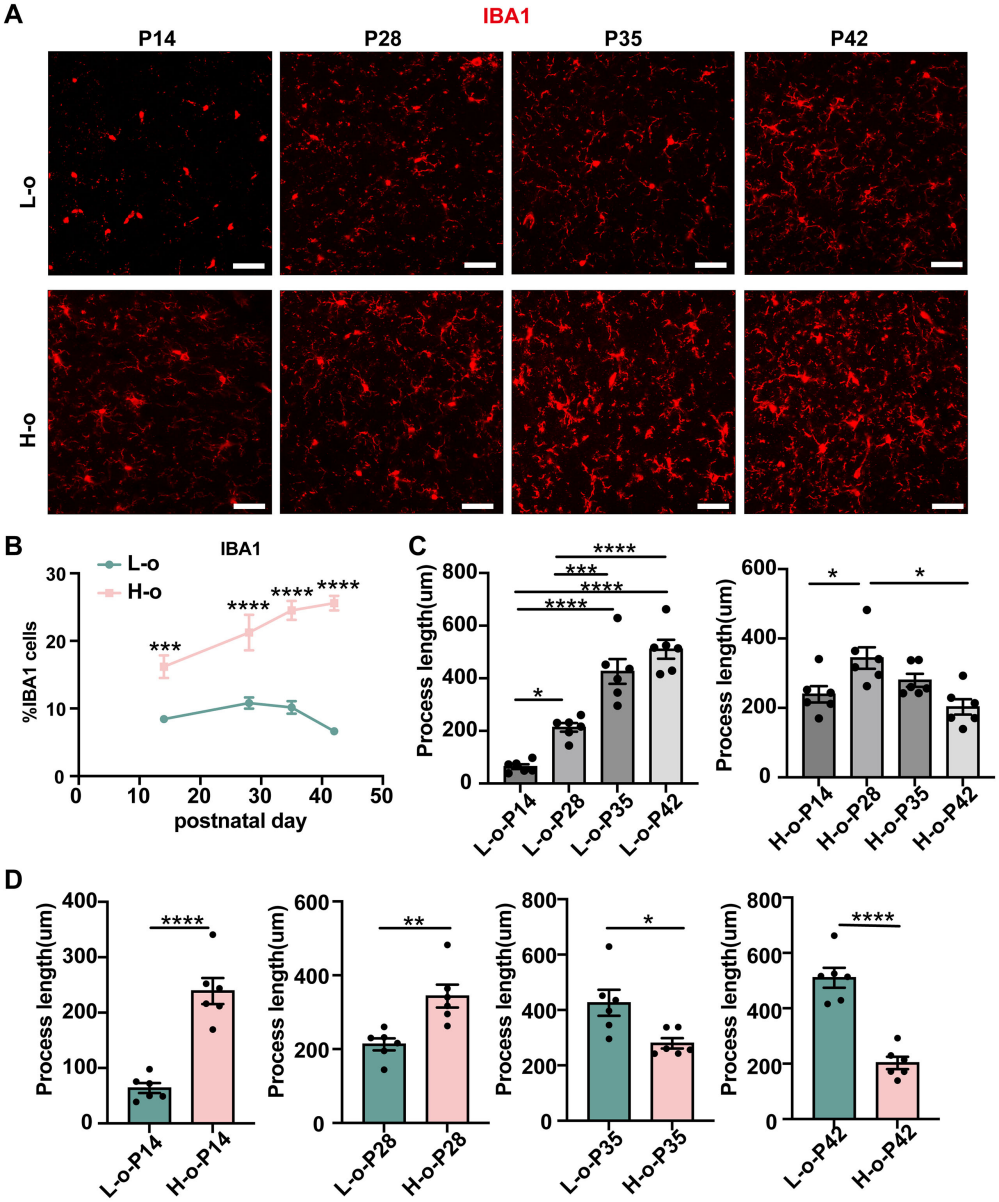
Morphological alterations of hypothalamic IBA1^+^ microglia-associated features in male offspring born to HFD-fed dams from postnatal to juvenile stages. **(A)** Representative immunofluorescence images illustrating IBA1^+^ microglia within the mediobasal hypothalamus (MBH) at P14, P28, P35, and P42 in male offspring born to dams fed a low-fat diet (L-o) or a high-fat diet (H-o). Images were shown at a magnification optimized for quantitative assessment of microglial processes across the MBH rather than detailed single-cell visualization. Scale bar, 75 µm. **(B)** Quantification of the proportion of IBA1^+^ cells in the MBH at P14, P28, P35, and P42. Each data point represented one litter-level biological replicate (n = 6 liters per group). **(C)** Temporal changes in the average process length of IBA1^+^ microglia across developmental stages in both groups (n = 6 liters per group). **(D)** Comparison of IBA1^+^ microglial process length between L-o and H-o offspring at P14, P28, P35, and P42 (n = 6 liters per group). Data were presented as mean ± SEM. Statistical analyses were performed using an unpaired two-tailed Student’s t test or two-way ANOVA followed by Sidak’s multiple-comparisons test. *p < 0.05, **p < 0.01, ***p < 0.001, ****p < 0.0001.

We next quantified IBA1^+^ microglial cells in the MBH across multiple postnatal stages (**Figure 3A**). In L-o mice, the number of microglia within the MBH gradually increased from P14 to P28, followed by a decline to lower levels by P42 (**Figure 3B**). Morphologically, these microglia transitioned from phagocytic, amoeboid-shaped cells lacking processes to ramified microglia characterized by progressively elongated and branched processes over time (**Figure 3C**). In contrast, H-o mice exhibited a significant increase in IBA1^+^ microglial density as early as P14, which persisted through P42 (**Figure 3B**). Although microglial branching appeared earlier in H-o than in L-o mice (**Figure 3D**; *p* < 0.0001), microglia in H-o mice progressively developed enlarged somata with thickened and shortened processes from P35 onward (**Figure 3D**; *p* = 0.016 at P35; *p* < 0.0001 at P42).

### 3.4 Reduced pSMAD3 immunoreactivity in IBA1-positive cells in male offspring born to HFD-fed dams during early postnatal life

**Figure 4:**
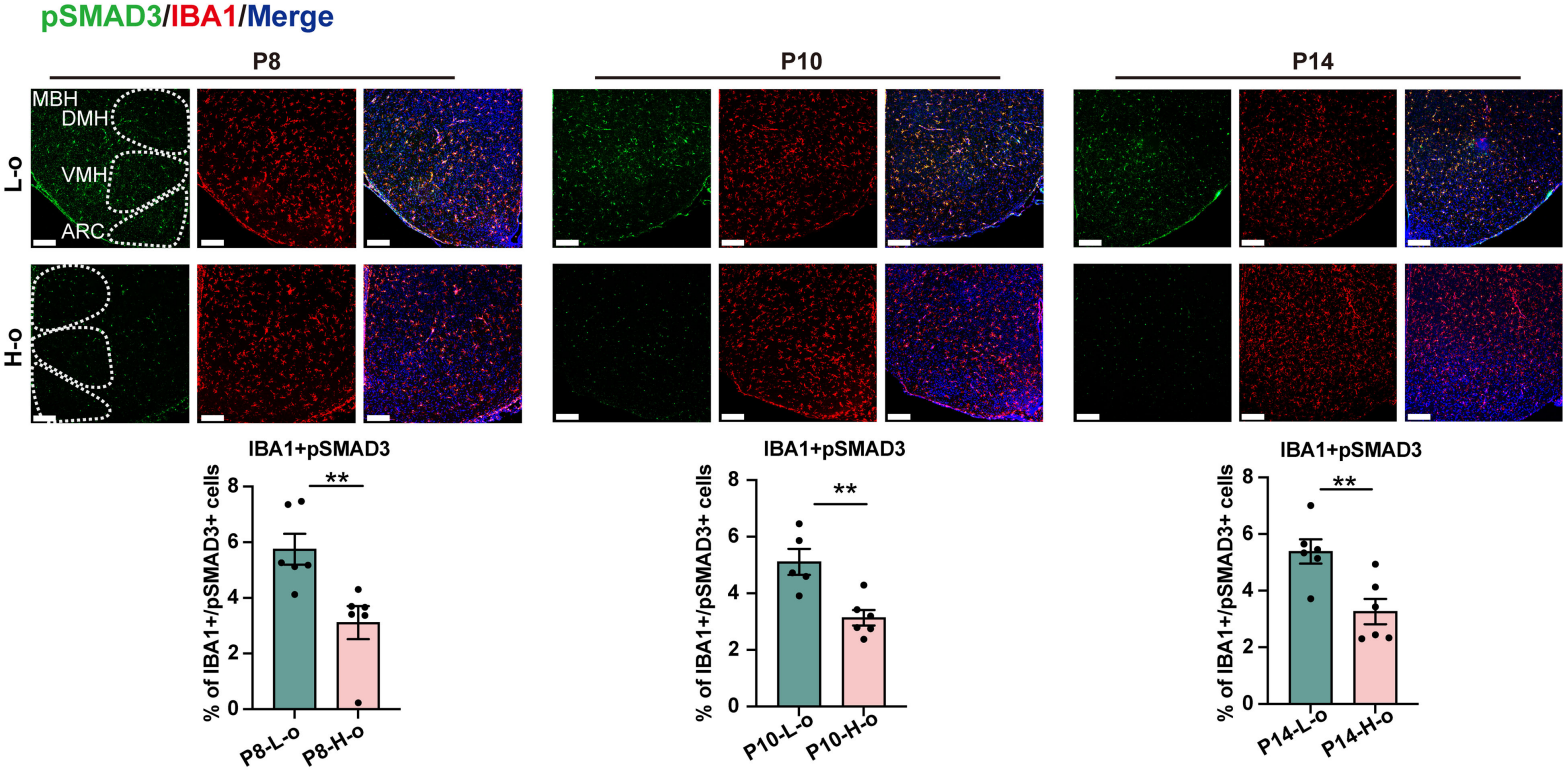
Reduced pSMAD3 immunoreactivity in IBA1^+^ cells within the hypothalamus during early postnatal development in male offspring born to HFD-fed dams. Representative low-magnification immunofluorescence images and quantification of the proportion of IBA1^+^/pSMAD3^+^ microglia in the mediobasal hypothalamus (MBH) at P8, P10, and P14 in male offspring born to dams fed a low-fat diet (L-o) or a high-fat diet (H-o). Each data point represented one litter-level biological replicate (P10 L-o: n = 5 liters; all other groups: n = 6 liters per group). Images were presented to illustrate region-specific patterns of pathway activation within the MBH rather than fine subcellular or single-cell morphological features. Scale bar, 150 µm. Data were presented as mean ± SEM. Statistical analyses were performed using an unpaired two-tailed Student’s t test or two-way ANOVA followed by Sidak’s multiple-comparisons test. **p < 0.01.

Previous studies have identified TGFβ1 signaling as an important regulator associated with acquisition of homeostatic microglial features during postnatal development (17). To assess potential changes in canonical TGFβ1 downstream signaling in H-o mice, we quantified pSMAD3 immunoreactivity in IBA1^+^ cells in the MBH at P8, P10, and P14. Across all stages examined, the proportion of IBA1^+^/pSMAD3^+^ cells were significantly lower in H-o than in L-o mice (**Figure 4**; P8: *p* = 0.0086; P10: *p* = 0.0040; P14: *p* = 0.0066), indicating persistently reduced pSMAD3 immunoreactivity in IBA1^+^ cells during early postnatal development. Given the established association between TGFβ1 signaling and developmental acquisition of homeostatic microglial characteristics reported in previous studies, the early postnatal reduction in pSMAD3 immunoreactivity observed in H-o mice temporally coincided with the later reduction in TMEM119^+^ cell abundance within the MBH.

### 3.5 Reduced Parkin-associated signal in hypothalamic microglia-defined regions of interest in male offspring born to HFD-fed dams during early postnatal life

**Figure 5:**
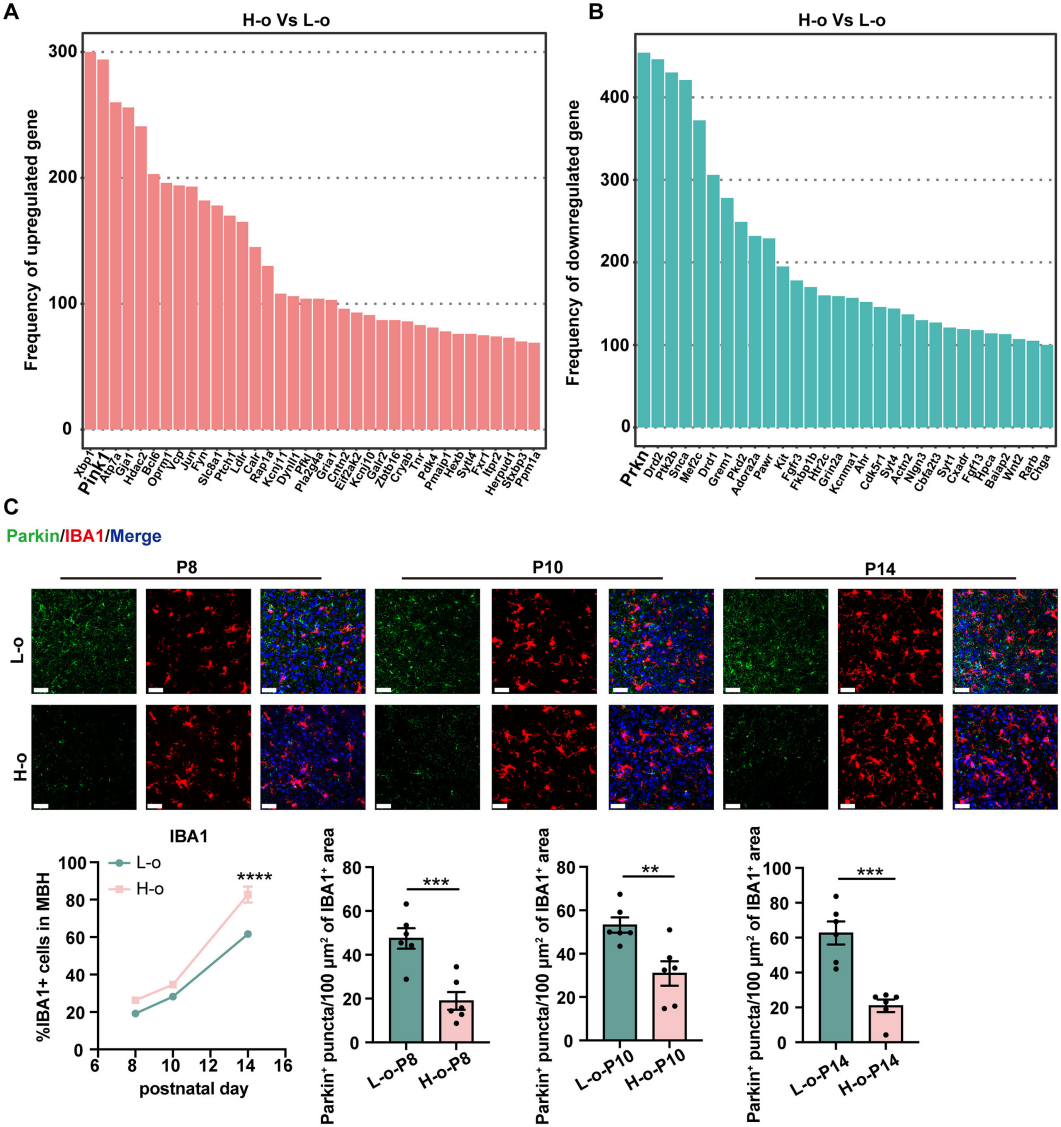
Reduced Parkin-associated puncta within IBA1-centered regions of interest in the hypothalamus of male offspring born to HFD-fed dams. **(A)** Frequency of genes appearing in significantly upregulated GO-enriched pathways from downstream analysis of bulk RNA-seq data of hypothalamic tissues at weaning in male offspring born to dams fed a low-fat diet (L-o) or a high-fat diet (H-o). **(B)** Frequency of genes appearing in significantly downregulated GO-enriched pathways from the same bulk RNA-seq dataset. **(C)** Representative immunofluorescence images and quantification showing the proportion of IBA1^+^ cells and the number of Parkin-associated puncta within standardized IBA1-centered regions of interest (ROI) in the mediobasal hypothalamus (MBH) at P8, P10, and P14. Each data point represented one litter-level biological replicate (n = 6 liters per group). Images were presented to illustrate regional signal patterns rather than definitive subcellular localization. Scale bar, 75 µm. Data were presented as mean ± SEM. Statistical analyses were performed using an unpaired two-tailed Student’s t test or two-way ANOVA followed by Sidak’s multiple-comparisons test. *p < 0.05, **p < 0.01, ***p < 0.001, ****p < 0.0001.

Differential expression analysis of RNA-seq data from the hypothalami of weaned L-o and H-o mice revealed a striking dysregulation of genes associated with mitochondrial quality-control pathways: Pink1 was among the most significantly upregulated, whereas Prkn was markedly downregulated (**Figures 5A, B**). Detailed DEG outputs and enrichment statistics were provided in **Supplementary Figure 2** and **Supplementary Table 2**. To assess whether this reduction is reflected within microglia-defined ROI in the MBH, we quantified Parkin immunoreactive puncta within standardized ROI(100 µm²) defined around individual IBA1^+^ microglia at P8, P10, and P14—a developmental window characterized by dynamic changes in microglial abundance and transcriptional states. Although microglial density increased in both groups, H-o mice exhibited a significantly higher microglial abundance by P14 (**Figure 5C**; *p* < 0.0001). Strikingly, across all three time points, the number of Parkin-associated puncta within standardized IBA1-centered ROI was significantly lower in H-o than in L-o mice (**Figure 5C**; P8: *p* = 0.0009; P10: *p* = 0.0072; P14: *p* = 0.0002).

### 3.6 Neonatal MBH TGFβ1 supplementation is associated with upregulation of local pSMAD3 signaling and subsequent changes in microglia-associated developmental features

**Figure 6:**
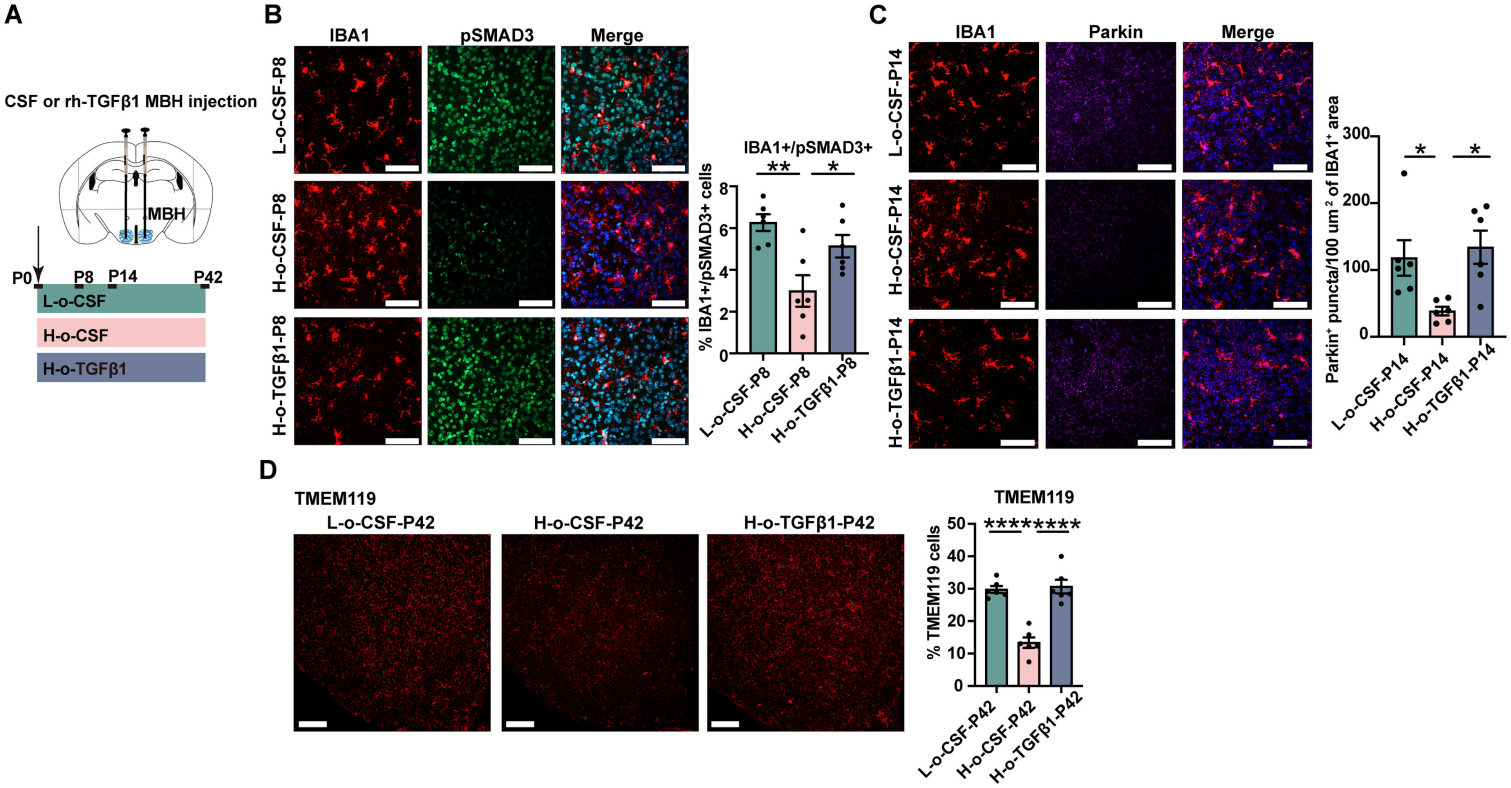
Neonatal MBH supplementation with recombinant TGFβ1 is associated with increased pSMAD3 immunoreactivity and increased TMEM119-positive cell abundance at later juvenile stages. **(A)** Schematic illustrating the stereotactically targeted bilateral injection sites for local neonatal TGFβ1 supplementation within the mediobasal hypothalamus (MBH) of neonatal mice and the experimental timeline. Three groups were included: L-o-CSF control, H-o-CSF control, and H-o-TGFβ1. **(B)** Representative immunofluorescence images and quantification of the proportion of IBA1^+^/pSMAD3^+^ cells primarily localized within the MBH at P8 (n = 6 liters per group). Scale bar, 75 µm. **(C)** Quantification of Parkin-associated puncta within standardized IBA1-centered regions of interest (ROI) in the MBH at P14 across the three experimental groups (n = 6 liters per group). Scale bar, 75 µm. **(D)** Quantification of TMEM119^+^ cells in the MBH at P42. Each data point represented one litter-level biological replicate (n = 6 liters per group). Scale bar, 150 µm. Data were presented as mean ± SEM. Statistical analyses were performed using one-way ANOVA followed by Tukey’s multiple-comparison test. *p < 0.05, **p < 0.01, ****p < 0.0001.

To test whether restoration of local early postnatal TGFβ1 signaling is associated with subsequent microglia-associated developmental features, we performed bilateral MBH microinjection of recombinant TGFβ1 (0.1 μg/ml, 1 μl per side), as optimized in pilot experiments in H-o neonates. The injection site is illustrated in **Figure 6A**. At P8, TGFβ1 supplementation significantly increased the proportion of IBA1^+^/pSMAD3^+^ cells in H-o mice (**Figure 6B**,;H-o-TGFβ1 vs H-o-CSF, *p* = 0.049), resulting in levels statistically comparable to L-o controls (**Figure 6B**; H-o-TGFβ1 vs L-o-CSF, *p* = 0.3833), accompanied by increased pSMAD3 immunoreactivity within the targeted region. At P14, the number of Parkin-associated puncta within standardized IBA1-centered ROI was significantly elevated following TGFβ1 treatment (**Figure 6C**; H-o-TGFβ1 vs H-o-CSF, *p* = 0.0166), reaching values indistinguishable from L-o mice (**Figure 6C**; H-o-TGFβ1 vs L-o-CSF, *p* = 0.8585). By P42, TMEM119^+^ cells were markedly increased in TGFβ1-treated H-o mice (**Figure 6D**; H-o-TGFβ1 vs H-o-CSF, *p* < 0.0001), again comparable to L-o controls (**Figure 6D**; H-o-TGFβ1 vs L-o-CSF, *p* = 0.9251). These findings indicate that restoration of local early postnatal TGFβ1 availability within the MBH is associated with subsequent increases in TMEM119^+^ cell abundance during later juvenile stages.

## 4 Discussion

In this study, we combined developmental single-cell reference analyses with temporally resolved immunohistochemistry to examine how maternal HFD exposure influences hypothalamic microglia-associated developmental features across postnatal life. Offspring from HFD-fed dams exhibited reduced pSMAD3 immunoreactivity in IBA1^+^ cells within the MBH during an early postnatal window. Across development, this was accompanied by dynamic changes in microglial abundance and morphology, as well as a blunted juvenile increase in TMEM119^+^ cell abundance within the MBH. Together, these findings indicate that maternal HFD exposure is associated with altered developmental trajectories of microglia-associated features during postnatal hypothalamic development.

Multiple lines of evidence have established TGFβ1 signaling as a central regulator of microglial development and the maintenance of homeostatic identity in the mature central nervous system (17, 27–30). During early postnatal life, TGFβ1 functions as a temporally restricted instructive cue associated with the acquisition of homeostatic-associated transcriptional programs in parenchymal microglia, predominantly through canonical SMAD2/3 signaling (17, 31). Genetic and developmental studies have demonstrated that disruption of TGFβ signaling during this sensitive window leads to long-lasting alterations in microglial identity (19), even in the absence of overt inflammation, underscoring the importance of appropriate pathway engagement for the establishment of homeostatic-associated microglial features during postnatal development.

Maternal HFD exposure is widely associated with chronic low-grade inflammation, elevated circulating pro-inflammatory cytokines, altered metabolic and endocrine signaling, and developmental neuroimmune perturbations during critical perinatal windows (32–36). In addition, accumulating evidence suggests that maternal HFD exposure may induce long-lasting epigenetic alterations within offspring hypothalamic developmental pathways, including changes affecting neuroimmune and metabolic signaling networks (35). Such developmental neuroimmune and epigenetic alterations may potentially contribute to altered local TGFβ1 signaling environments and impaired acquisition of homeostatic marker-associated features during critical postnatal windows.

In this context, the pronounced reduction of pSMAD3 observed in the hypothalamus of offspring born to HFD-fed dams during early postnatal life is most consistent with insufficient engagement of canonical TGFβ signaling during a critical developmental period, rather than a compensatory or anti-inflammatory response. Indeed, compensatory increases in TGFβ activity are more commonly described in acute injury or inflammatory settings, where enhanced signaling acts to restrain excessive microglial activation and cytokine production (37, 38). The developmental timing, regional specificity, and directionality of the pSMAD3 changes observed here suggest that maternal HFD exposure may influence developmental processes associated with subsequent changes in TMEM119^+^ cell abundance and homeostatic marker-associated features within the MBH. Consistent with this interpretation, neonatal MBH-targeted supplementation of TGFβ1 restored pSMAD3 immunoreactivity toward levels observed in control offspring and was associated with a partial normalization of TMEM119^+^ cells abundance at later juvenile stages. Importantly, the neonatal TGFβ1 supplementation paradigm should be interpreted as restoration of a locally deficient developmental signaling environment within the MBH rather than as a microglia-specific intervention. Because multiple MBH-resident cell populations may respond to TGFβ1 during early postnatal development (39), the present study cannot distinguish direct microglial effects from indirect effects mediated through broader hypothalamic cellular interactions. Accordingly, our conclusions are limited to demonstrating that restoration of early postnatal TGFβ1 availability within the MBH is associated with alterations in microglia-associated developmental features under maternal HFD exposure. Regional specificity of the intervention was inferred from the localized increase in pSMAD3 immunoreactivity observed within the MBH following neonatal TGFβ1 administration. However, molecular diffusion beyond the injection site was not systematically assessed using tracer-based approaches. Furthermore, while no overt tissue disruption or gross histological abnormalities were observed, acute injection-induced inflammatory activation, including potential alterations in microglial activation state or inflammatory cytokine expression, was not systematically evaluated. Therefore, subtle secondary cellular effects associated with the injection procedure cannot be completely excluded.

In parallel with suppressed canonical TGFβ1–SMAD3 signaling, we observed a consistent reduction in Parkin-associated signal within the hypothalamus and within microglia-defined regions of interest during early postnatal life. Parkin is widely recognized as a component of mitochondrial quality-control pathways (40), but its expression and punctate localization alone cannot be equated with functional mitophagic flux, particularly in the absence of dynamic or ultrastructural readouts (41). Accordingly, we do not interpret these findings as direct evidence of impaired mitophagy. Instead, we view the reduction in Parkin-associated signal as a microglia-associated molecular feature that parallels diminished canonical TGFβ signaling during a critical developmental window. Emerging evidence suggests that TGFβ pathways can influence mitochondrial homeostasis and stress-response programs in a context-dependent manner, including indirect modulation of mitochondrial regulatory proteins (42, 43). In this developmental setting, reduced Parkin expression may therefore reflect a broader alteration in developmental microglia-associated cellular programs downstream of insufficient TGFβ1–SMAD3 engagement, rather than a discrete defect in mitochondrial clearance. Notably, neonatal MBH-targeted TGFβ1 supplementation was accompanied by increased pSMAD3 immunoreactivity in IBA1^+^ cells and increased Parkin-associated signal within microglial ROIs, together with partial recovery of TMEM119-associated homeostatic-like features at later juvenile stages. While these parallel changes do not establish a direct mechanistic link between TGFβ signaling and Parkin regulation, they support the concept that early postnatal TGFβ1 availability influences coordinated developmental changes associated with homeostatic-like microglial features within the MBH. Determining whether mitochondrial quality-control pathways actively contribute to this process will require future studies incorporating microglia-enriched transcriptomics, mitophagy flux assays, and functional mitochondrial analyses.

Several limitations of the present study should be acknowledged. First, the public single-cell RNA sequencing dataset used in this study was derived from wild-type hypothalamic tissue rather than offspring exposed to maternal HFD. Accordingly, these analyses were primarily intended to provide a developmental reference framework for normal postnatal hypothalamic microglial dynamics and to guide the selection of postnatal observation windows for subsequent immunohistochemical analyses. Recent consensus perspectives further suggest that postnatal microglia exhibit highly dynamic and heterogeneous transcriptional states across developmental windows (44), supporting the importance of temporally resolved analyses during early hypothalamic maturation. Nevertheless, the developmental trajectories and microglial subcluster distributions identified in wild-type mice may differ from those present under maternal HFD exposure, and the current study does not directly resolve microglia-intrinsic transcriptional alterations within the model itself. Furthermore, because microglia-specific transcriptomic validation was not performed in our experimental cohorts, the extent to which maternal HFD exposure alters intrinsic developmental microglial programs versus indirectly reshapes microglial states through altered intercellular signaling within the MBH remains to be fully resolved.

Second, TMEM119^+^ cells were quantified relative to total DAPI^+^ nuclei within the MBH rather than relative to a microglia-defined denominator such as total IBA1^+^ cells. Although this normalization strategy was adopted to account for dynamic developmental changes in tissue size and overall cellular composition across postnatal stages, it does not fully distinguish alterations in TMEM119-associated homeostatic-like features from broader changes in microglial abundance or local tissue architecture. In addition, because TMEM119/IBA1 colocalization analyses were not performed, TMEM119-associated signals should be interpreted cautiously as developmental microglia-associated features rather than definitive evidence of intrinsic microglial maturation states.

Finally, the present study exclusively examined male offspring. Although male offspring were selected based on previous evidence demonstrating earlier and more pronounced metabolic abnormalities under maternal HFD exposure (22, 45), microglial development and neuroimmune adaptation are known to exhibit important sex-dependent characteristics (46, 47). Therefore, the present findings should be interpreted specifically within the context of male offspring, and future studies incorporating female offspring will be necessary to more comprehensively define sex-dependent hypothalamic microglial developmental trajectories under maternal HFD exposure.

The translational implications of these findings also warrant consideration. Direct investigation of fetal or early postnatal human microglial programming remains highly challenging because microglia are largely inaccessible during critical developmental windows. Recent studies have therefore proposed several human-accessible systems for modeling early-life neuroimmune development, including iPSC-derived microglia (48), PBMC-derived microglia-like cells (49), brain organoids (50), and placental macrophages (Hofbauer cells) (33). Notably, fetal placental Hofbauer cells and fetal brain microglia have been shown to share overlapping transcriptional programs and similar responses to maternal diet-induced obesity, raising the possibility that placental immune profiling at birth may provide a clinically accessible surrogate window into fetal microglial programming (51). These findings raise the possibility that placental immune profiling at birth may provide a clinically accessible surrogate window into fetal microglial programming under maternal metabolic stress conditions. Although the present study was conducted exclusively in a mouse model, our findings are consistent with the broader concept that early-life neuroimmune developmental alterations induced by maternal HFD exposure may contribute to long-term offspring metabolic and neurodevelopmental vulnerability. Future studies integrating human placental immune profiling with spatial transcriptomic and stem cell–based neuroimmune modeling approaches may help bridge experimental rodent findings with human developmental neuroimmunology.

Together, these data support a developmental model in which maternal HFD exposure is associated with reduced canonical TGFβ/SMAD pathway engagement within the MBH during a critical postnatal window, coinciding with altered TMEM119^+^ cells abundance and microglia-associated developmental features. Although metabolic outcomes were not directly assessed in the present study, previous studies using maternal HFD exposure models have consistently demonstrated increased susceptibility to hyperphagia, impaired glucose homeostasis, and altered hypothalamic feeding-circuit development in offspring (4, 52, 53). Therefore, the developmental alterations described in the present study may represent candidate neuroimmune mechanisms potentially associated with these previously reported metabolic phenotypes. Nevertheless, because these outcomes were not directly measured here, such links should be considered hypothesis-generating and will require direct functional validation in future studies.

## Data Availability

The original contributions presented in the study are included in the article/**Supplementary Material**. Further inquiries can be directed to the corresponding author.
